# A new strategy for controlling invasive weeds: selecting valuable native plants to defeat them

**DOI:** 10.1038/srep11004

**Published:** 2015-06-05

**Authors:** Weihua Li, Jianning Luo, Xingshan Tian, Wah Soon Chow, Zhongyu Sun, Taijie Zhang, Shaolin Peng, Changlian Peng

**Affiliations:** 1 Guangdong Provincial Key Laboratory of Biotechnology for Plant Development, Key Laboratory of Ecology and Environmental Science in Guangdong Higher Education, School of Life Science, South China Normal University, Guangzhou, 510631 China; 2 Vegetable Research Institute, Guangdong Academy of Agricultural Sciences, Guangzhou, 510640 China; 3 Plant Protection Institute, Guangdong Academy of Agricultural Sciences, Guangdong Provincial Key Laboratory of High Technology for Plant Protection, Guangzhou 510640, China; 4 Division of Plant Science, Research School of Biology, College of Medicine, Biology and Environment, The Australian National University, ACTON, Australian Capital Territory 2601, Australia; 5 State Key Laboratory of Bio-control, Sun Yat-Sen University, Guangzhou, 510275 China

## Abstract

To explore replacement control of the invasive weed *Ipomoea cairica*, we studied the competitive effects of two valuable natives, *Pueraria lobata* and *Paederia scandens*, on growth and photosynthetic characteristics of *I. cairica*, in pot and field experiments. When *I. cairica* was planted in pots with *P. lobata* or *P. scandens*, its total biomass decreased by 68.7% and 45.8%, and its stem length by 33.3% and 34.1%, respectively. The two natives depressed growth of the weed by their strong effects on its photosynthetic characteristics, including suppression of leaf biomass and the abundance of the CO_2_-fixing enzyme RUBISCO. The field experiment demonstrated that sowing seeds of *P. lobata* or *P. scandens* in plots where the weed had been largely cleared produced 11.8-fold or 2.5-fold as much leaf biomass of the two natives, respectively, as the weed. Replacement control by valuable native species is potentially a feasible and sustainable means of suppressing *I. cairica*.

Invasion by exotic weed species is a serious threat to natural ecosystems. Attempts have been made to seek economical and sustainable methods to reduce the abundance and dominance of noxious weeds for many years^1^,^2^,^3^. Control of invasive species can be achieved through mechanical or chemical methods and also through biocontrol agents^4^. Although the value of biological control is evident and attractive^5^,^6^, the evaluation of potential biocontrol insects is a long and very costly process, so mechanical control and chemical herbicides have been most often used in weed management. Mechanical removal by cutting or chemical control by spraying herbicides can rapidly suppress the weeds, but they can easily regenerate. The urgent question is: if the vegetation canopy is opened up after the reduction of the weed coverage by any control method, what could fill the resulting gaps to hamper the re-establishment of the weed canopy?

Some studies have proposed that fast-growing trees could be selected to restructure the community invaded by the vine, *Mikania micrantha*, since the restructured community can suppress the recruitment of *M. micrantha* for the long term^7^,^8^. The recruitment of an invasive vine *Macfadyena unguis*-*cati* was prevented when the two perennial shrubs, *Syngonium podophllum* and *Cuphea hyssopifolia*, were planted to occupy the empty space and to form a dense canopy after *M. unguis-cati* was removed^9^. However, interspecific competition should be greatest between functionally similar species based on the principle of limiting similarity^10^,^11^,^12^; thus, selecting native plants with the same life form as the invader will more effective. However, the efficacy of planting vines to control vines is not clear.

*Ipomoea cairica* (L.) Sweet (Convolvulaceae) is an extremely fast growing, sprawling, perennial liana, believed to have originated from a rather wide area: Africa, Asia, Pacific Islands and South America^13^. It is recognized as the second worst invasive weed in southern China following * M. micrantha*^14^,^15^, occurring widely in thickets, roadsides, waste places, cultivated areas and sunny meadows in Guangdong, Guangxi, Hainan, Fujian, Taiwan and Yunnan^13^,^14^. It forms extensive monocultures which transform natural habitats, and is problematic in parks, forests, plantations, orchards and tea and nursery gardens^16^,^17^.

We observed that *Pueraria lobata* and *Paederia scandens* often coexist with *I. cairica* in the same habitats in the field and can be considered as belonging to the same functional group. *P. lobata* (kudzu vine) is a climbing perennial vine native to China and widely distributed from Southeast Asia to Australia. Its vegetative growth can be very rapid (up to 26 cm per day or 15 m per growing season) and plants produce new roots where nodes contact soil. It has various positive uses. For example, its roots can yield extracted starch as food or can be harvested for medicinal use, its leaves and shoots can be nutritious forage for livestock, and it is also used for land improvement through nitrogen fixation and prevention of erosion^18^. *P*. *scandens* (Chinese feverine), native to China, is also a rapidly growing vine, widely distributed in provinces south of Chang Jiang (the Yangtze River) as well as in the riverside areas. This plant has medicinal properties and a decoction of the whole plant is used in the treatment of abdominal pain, abscesses, arthritis, over-eating and more^19^. Its leaves are edible and used for making a Chinese traditional health food named Chinese Feverine rice cake, which is very popular in the countryside of South China.

Apart from the characteristics mentioned-above, the two native species, particularly *P. lobata*, have traits that help to intercept more light such as longer main stems (i.e. taller plants), larger leaf areas and larger above-ground biomass per plant, compared with *I. cairica*. These traits play an important role when there is competition for light because larger individuals may reduce the light available to smaller individuals and thus suppress their growth^20^. Furthermore, past studies of photosynthesis associated with the invasive species, *I. cairica,* have mostly focused on the plant itself growing alone, suggesting that *I. cairica* is intrinsically a helophytic plant^15^. However, little is known about changes in photosynthetic characteristics when *I. cairica* competes with other plants.

Therefore, we selected the two valuable native lianas to fill the gaps in the community where *I. cairica* coverage has been reduced, expecting that the structure and function of the native ecosystem could be re-established. We conducted one outdoor pot experiment and one field experiment (to further examine the results of the pot experiment) to investigate (a) how the native species would compete with the alien weed and which one would dominate, and (b) changes in photosynthetic characteristics when the native plants competed with the invasive species. We wanted to test the hypotheses: (1) *P. lobata* and *P. scandens* are superior competitors to *I. cairica*. We expected the effects of the two native species on the alien to be stronger than that of the alien on the native species. We also expected the natives to be less affected by interspecific or intraspecific competition than the alien. (2) Replacement control through planting the valuable native species could prevent recruitment of the invasive weed, *I. cairica*.

## Results

### Changes in plant growth and relative interaction index in a pot experiment

When the three plants were grown separately, the total biomass of each of the two native species was significantly greater than that of the invasive species, with *P. lobata* having by far the highest biomass among them (Table 1). The total biomass of *I. cairica* grown in competition with *P. lobata* was significantly lower than that under intraspecific competition or when it was growing alone (Table 1). Conversely, the total biomass of *P. lobata* in competition with *I. cairica* was significantly greater than that under intraspecific competition, though lower than that when *P. lobata* was growing alone (Table 1). There was no significant difference between the total biomass of *P. scandens* when it was grown with *I. cairica* and when it was under intraspecific competition (Table 1). The caulis length showed similar trends as total biomass, except that *I. cairica* had significantly greater caulis length when it grew alone compared to other treatments (Table 1). Furthermore, as compared with intraspecific competition, the root mass ratio (RMR) of *I. cairica* increased significantly when it was planted with one of the two native species, whereas the root mass ratio (RMR) of *P. lobata* decreased significantly when it was planted with *I. cairica* or *P. scandens* (Table 1).

**Figure 1:**
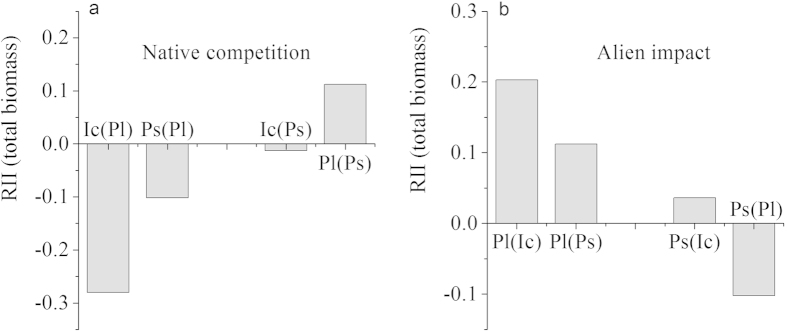
A comparison of the relative interaction index (RII) for quantifying native species competition and alien species impact. Ic(Pl) means the target plant was *I. cairica* growing with *P. lobata* under interspecific competition; Ps(Pl) means the target plant was *P. scandens*, growing with *P. lobata* under interspecific competition; Ic(Ps) means the target plant was *I. cairica* growing, with *P. scandens* under interspecific competition; Pl(Ps) means the target plant was *P. lobata*, growing with *P. scandens* under interspecific competition; Pl(Ic) means the target plant was *P. lobata*, growing with *I. cairica* under interspecific competition; Ps(Ic) means the target plant was *P. scandens*, growing with *I. cairica* under interspecific competition.

Interactions between plants, in general, consisting of competition and facilitation, can be described by the relative interaction index (RII, defined in Methods)^21^. RII has values ranging from −1 to +1 and it is symmetrical around zero. A negative value indicates competition and a positive value indicates facilitation. Fig. 1a shows that the RII of *I. cairica* grown in the presence of *P. lobata* or *P. scandens* was negative (RII_Ic(Pl)_ = −0.280, RII_Ic(Ps)_ = −0.013, respectively), indicating that the two native species competed well with the alien species, and that the magnitude of the negative effect of *P. lobata* on *I. cairica* was larger than that of *P. scandens* on *I. cairica* (0.280 > 0.013). That is, *P. lobata* was a stronger competitor than *P. scandens* against *I. cairica*. Further, the effect of *P. scandens* on *P. lobata* was positive (RII_Pl(Ps)_ = 0.112) (Fig. 1a), indicating that *P. scandens* could promote the growth of *P*. *lobata*, relative to growth in conditions of intraspecific interaction.

Regarding the impact by an alien species, *I. cairica* had a positive effect on *P. lobata* and *P. scandens* [RII_Pl(Ic)_ = 0.203, RII_Ps(Ic)_ = 0.036] (Fig. 1b), indicating that *I. cairica* could promote the growth of the two native species (relative to intraspecific interaction conditions). The effect of *I. cairica* on *P. lobata* was more positive than that of *P. scandens* on *P. lobata* (Fig. 1b) and *P. lobata* had a relatively small negative effect on *P. scandens* (Fig. 1a), indicating that the facilitation by the alien species was larger than the impact that the two natives had on each other.

### Changes in gas exchange parameters and chlorophyll fluorescence parameters in a pot experiment

**Figure 2:**
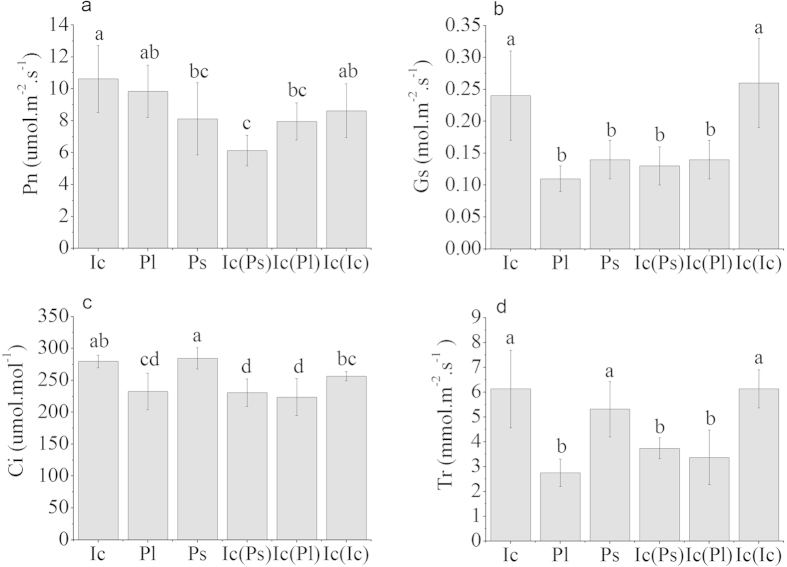
Changes in gas exchange parameters (means ± SD, n = 6). Ic, Pl and Ps means *I. cairica*, *P. lobata* and *P. scandens* growing alone respectively; Ic(Ps) and Ic(Pl) means *I. cairica* growing with *P. scandens* and *P. lobata*, respectively, under interspecific competition; Ic(Ic) means *I. cairica* under intraspecific competition. *P*_*n*_ - the net photosynthetic rate, *G*_*s*_ - stomatal conductance, *C*_*i*_ - the intercellular CO_2_ concentration, and *T*_*r*_ - the transpiration rate. Different letters above columns indicate significant differences between competition treatments according to the Least-Significant Difference test (LSD-test, *P* < 0.05).

When *I. cairica* grew alone or under intraspecific competition, the gas exchange parameters were often greater than those of *I. cairica* growing with *P. lobata* or *P. scandens* (Fig. 2), showing the superior photosynthetic ability of the invasive species in the absence of interspecific competition. The net photosynthetic rate *P*_*n*_, stomatal conductance *G*_*s*_, the intercellular CO_2_ concentration *C*_*i*_ and the transpiration rate *T*_*r*_ decreased significantly when *I. cairica* was grown under interspecific competition with *P. lobata* or *P. scandens* (Fig. 2).

When *I. cairica* and *P. lobata* were grown alone, there were no significant differences between them in the maximal photochemical efficiency (*F*_*v*_/*F*_*m*_) of dark-relaxed Photosystem II (the photosystem that splits water to evolve oxygen), the electron transport rate estimated by chlorophyll fluorescence (ETR) and the photochemical yield of Photosystem II under illumination (*Φ*_*PSII*_). However, when *I. cairica* was grown with one of the two native species under interspecific competition, *F*_*v*_/*F*_*m*_, ETR and *Φ*_*PSII*_ decreased significantly, being lower than when *I. cairica* was grown alone or under intraspecific competition (Table 2). Similarly, *I. cairica* grown with *P. scandens* or *P. lobata* exhibited a significantly lowered content of Rubisco (the enzyme complex that fixes CO_2_) in the plant’s leaves when compared to *I. cairica* under intraspecific competition or when grown alone (Table 2). On the other hand, there was no significant difference in stomatal limitation between interspecific and intraspecific competition treatments. It appears that the lower net photosynthetic rate of *I. cairica* under interspecific competition could be attributed to a decrease in Rubisco content but not to a stomatal limitation factor.

### Effects of replacement control in the field

**Figure 3:**
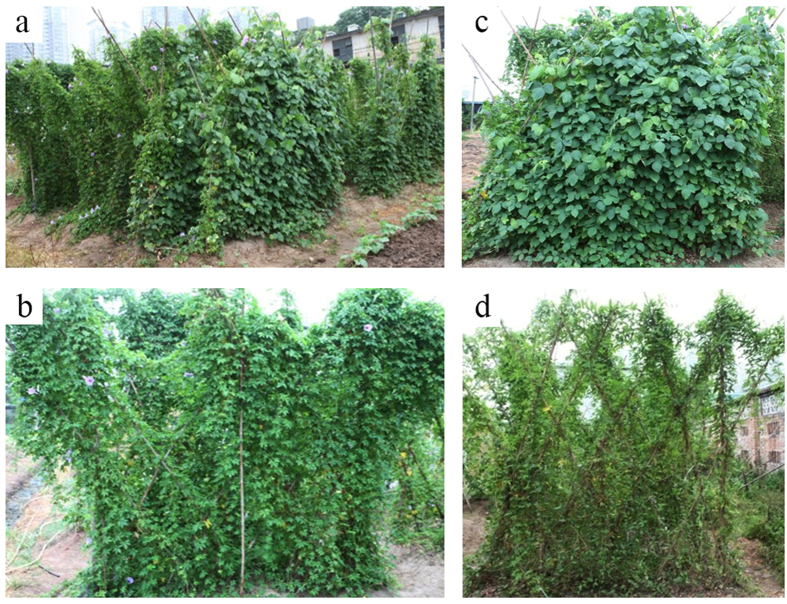
Pictures of the experimental field (**a** - the whole experiment plots; **b** – no replacement plots as the control; **c** – the plots replaced by *P. lobata*; **d** – the plots replaced by *P. scandens*).

Five months after sowing seeds of the two native species, the whole experiment plots were pictured in Fig. 3a. There was little recruitment of the invasive weed *I. cairica* in the plots replaced by the two native species (Fig. 3c,d). By contrast, there was much recruitment of *I. cairica* in the control plots (Fig. 3b), from which the aboveground biomass of *I. cairica* and most roots had been cleared five months before.

The biomass of roots, stems, leaves and flowers of the invasive species *I. cairica* decreased significantly in the plots replaced by the two native species, compared to the control plots (Table 3). *P. lobata* had the greatest total biomass: its aboveground biomass was considerably greater than that of *I. cairica* in any treatment, and its foliar biomass, in particular, was 2.2 times that of *I. cairica* in the control plots, though its root biomass was much lower (Table 3). The caulis length of *I. cairica* also decreased significantly in the plots replaced by native species compared with the control plots. Replacement control caused a great reduction in total biomass and caulis length of the invasive species (Table 3).

**Figure 4:**
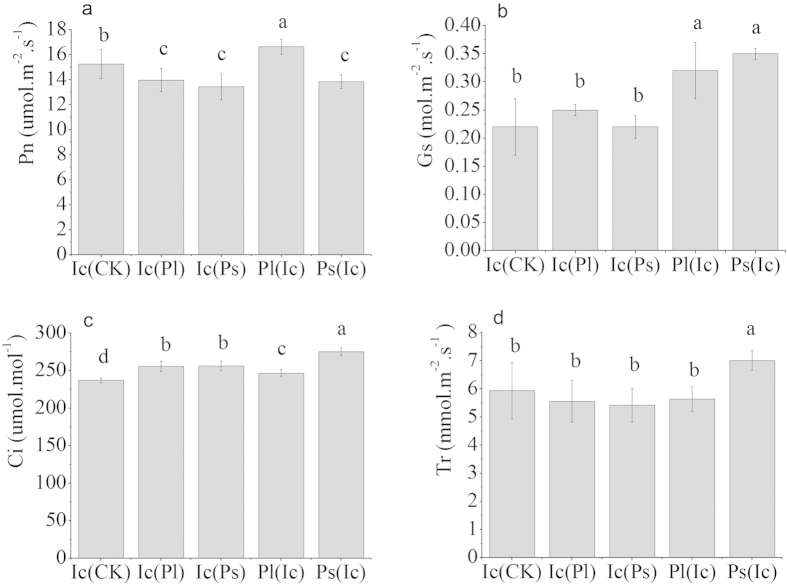
Changes of gas exchange parameters in plants in the field experiment (means ± SD, n = 6). Ic(CK) means *I. cairica* recruiting in the control plots not replaced by any native species; Ic(Pl) means *I. cairica* recruiting in the plots replaced by the native species *P. lobata*; Ic(Ps) means *I. cairica* recruiting in the plots replaced by the native species *P. scandens*; Pl(Ic) means *P. lobata* in the same plots as Ic(Pl); Ps(Ic) means *P. scandens* in the same plots as Ic(Ps). *P*_*n*_ - the net photosynthetic rate, *G*_*s*_ - stomatal conductance, *C*_*i*_ - the intercellular CO_2_ concentration, and *T*_*r*_ - the transpiration rate. Different letters above columns indicate significant differences between competition treatments according to the Least-Significant Difference test (LSD-test, *P* < 0.05).

Replacement control also led to a decline in the net photosynthetic rate (*P*_*n*_) of *I. cairica* in the field (Fig. 4a). Compared with that in the control plots, the intercellular CO_2_ concentration (*Ci*) of *I. cairica* under interspecific competition showed the reverse trend (Fig. 4c). Stomatal conductance (*G*_*s*_) and the transpiration rate (*T*_*r*_) of *I. cairica* were not significantly affected by competition from the two native species (Fig. 4b,d).

Changes in soil chemical characteristics showed that soil fertility improved in *P. lobata* and *P. scandens* plots. Total nitrogen (TN), NH_4_-N and soil organic matter (SOM) increased significantly in *P. lobata* and *P. scandens* plots compared with those in the control plots, highest in the nitrogen fixer, *P. lobata*, plots (Table 4).

## Discussion

Interspecific competition has been reported to play an important role in determining the likelihood of success in the replacement control of invasive weeds^22^,^23^. However, when testing the hypothesis that a native species is a better competitor than an invasive species, simultaneous consideration of both the relative competitiveness of a native species against the invader, and the invader’s relative impact on the native species has rarely been attempted^24^. If a native species is to be competitive, we expect it to reduce the growth of the invasive species, *I. cairica*, more than it could reduce the growth of another coexisting native. Indeed, this was observed in the pot experiments: the native species *P. lobata* significantly reduced *I. cairica* growth (RII_Ic(Pl)_ = −0.280), while the competition between the two natives gave positive or less negative RII values (Fig. 1a). With regard to the invader’s relative impact, we expected that the negative effect of the invader on the natives would be less than that of the natives on the invader. Indeed, this was the outcome: *I. cairica* facilitated the growth of the two natives [RII_Pl(Ic)_ = 0.203 and RII_Ps(Ic)_ = 0.036] relative to growth under intraspecific competition (Fig. 1b). Therefore, at the level of the individual, the two native species have the potential to replace the invasive species *I. cairica*, with *P. lobata* having the greater control potential than *P. scandens*.

What underpins the competitiveness of the two native species, particularly *P. lobata*? Changes in the root mass ratio of *P. lobata* indicated that less biomass was allocated to roots and more biomass was allocated to shoots when *P. lobata* was in competition with *I. cairica* or *P. scandens* (Table 1). By contrast, the root mass ratio of *I. cairica* increased when the invasive weed was in competition with the two native species, as compared with growth in intraspecific competition conditions in the pot experiment (Table 1). Similarly, comparing Ic(Pl) and Pl(Ic) in the field experiment in Table 3, while the root biomass was similar, *P. lobata* had 11-fold more leaf biomass per plant, and five-fold more stems, compared with *I. cairica*. Similarly, *P. scandens* had 2.5-fold more leaf biomass and 5.5-fold more stems compared with *I. cairica* in interspecific competition conditions. Together, these effects imply that the two native species invested more biomass in light interception, thereby increasing total photosynthetic productivity.

Another factor that lowers the competitiveness of the invasive species, *I. cairica*, is that interspecific competition reduced its rate of photosynthesis per unit leaf area (Fig. 2), accompanied or caused by a decrease in Rubisco content (Table 2). Gas exchange parameters in the field experiment also showed that *I. cairica* had a lower *P*_*n*_ in the presence of competition from the native species (Fig. 4a). Perhaps the competition for light resulted in partial shading of the *I. cairica* leaves by *P. lobata* or *P. scandens* leaves. A slightly lower growth irradiance to which *I. cairica* leaves were exposed would represent a lower-light environment which would give rise to a lower content of cytochrome *bf* (often a rate-limiting bottle-neck in electron flow from PS II to PS I) and a lower Rubisco content^25^. Since *I. cairica* has relatively high light requirements^15^, reduced light levels due to crowding could be the main reason for its reduction in photosynthetic rates in the presence of interspecific competition.

Another possible reason for the much greater amount of aboveground biomass of *P. lobata* growing under interspecific competition with *I. cairica* is its ability to fix atmospheric nitrogen^18^. Indeed, the total soil nitrogen was almost 10-fold higher in the *P. lobata* plot compared with the *I. cairica* plot (Table 4). *P. scandens* enriched soil nitrogen to an immediate extent, lower than that in the *P. lobata* plot (Table 4), probably because it is not a nitrogen-fixing plant. Its total biomass in a pot experiment was also intermediate [comparing Ic(Ic), Pl(Ic) and Ps(Ic) under competition conditions in Table 1], though this was not the case in the field experiment (Table 3). All else being equal, using a native legume is a better option for the replacement control of an invasive weed.

Our results are consistent with another study of the competition effects between the native grass, *Imperata cylindrica* (Poaceae), and the invasive herb, *Ageratina adenophora* (Asteraceae). *I. cylindrica* had a higher competitive ability than *A. adenophora*, being able to heavily suppress the growth of *A. adenophora* by shoot competition^26^. Another example is the seedling competition between native cottonwood and exotic saltcedar; when native plants have rapid seedling establishment, they can compete with invasive weeds in re-vegetation projects^1^. Both *Imperata cylindrica* and *Ageratina adenophora* are herbaceous plants, while cottonwood and saltcedar are trees, each pair having the same life form, just as vine *versus* vine in our study.

Niche-based community assembly theory predicts that communities should be resistant to invasion by non-native species if they contain native species that have traits similar to the common non-natives^27^,^28^,^29^,^30^. In restoration, this concept may guide the selection of native plants, supporting the use of natives with traits similar to those of invaders^10^,^31^, since a resident species whose niche overlaps with that of an invading species will compete most effectively with the invader^32^,^33^. Therefore, we suggest that selection of a similar life form, sympatric congeners or the same habitat with the invasive plants should be regarded as the preferred option when choosing plant species to replace invasive species. Economic value and ecological security should also be considered, such that economically valuable native species should be given priority. Moreover, if the chosen native species have high seed yields, as is the case of *P. lobata* and *P. scandens* here^18^,^19^, easy and simple sowing methods will help in replacing the invasive species in the field. Replacement control does not result in environmental pollution or re-sprouting of the weeds as do chemical herbicides or mechanical removal, and it offers a safe, economical, and environmentally sustainable solution for weed management.

In conclusion, we have demonstrated that replacement control through planting valuable native species can be a potential means of preventing the invasive weed *I. cairica* from re-growing. Our results showed that the impact of a one-off replacement control was significant in the short term (about half a year). Further studies need to be conducted on the succession results of replacement control in the long term so as to provide a complete understanding of the ecological restoration of the invaded habitats.

## Materials and Methods

### Culture of plant materials in a pot experiment

Seeds of *P. lobata* and *P. scandens* were collected from the campus of South China Normal University at the end of 2008 (lat. 28°08′N, long. 113°09′E, elevation 65 m above sea level). In March 2009, seeds of the two native vines were sown in flat trays and put in an artificial climate incubator (day: 30 °C, 12 h, 65% humidity; night: 23 °C, 12 h, 50% humidity) to germinate before transplanting. Because of the extremely low production amount and viability of *I. cairica* seeds, most of its spread in China is due to vegetative growth. Therefore, *I. cairica* rhizomes collected in the Biological Garden at South China Normal University were selected as the experimental materials. To ensure that all material was of similar sprouting potential, rhizomes with similar diameter and of the same age were cut into 10 cm-long fragments, on which there were at least two nodes. Cuttings were grown in plastic cups (diameter 7 cm, height 8 cm) filled with sand, one cutting per cup, and watered every two days and fertilized with 100% Hoagland’s nutrient solution once a week before transplanting.

### Competition treatment in a pot experiment

In April 2009, three weeks after sowing and sprouting, seedlings of *P. lobata* and *P. scandens*, and the regenerated plantlets of *I. cairica* were transplanted outdoors into pots (diameter 18 cm, height 16 cm) filled with soil (pond mud:sand:humus = 1:1:1) at a naturally-lit experimental site in the Biological Garden from where the founding rhizome had originated. Nine competition treatments which included all possible pair-wise combinations of intraspecific and interspecific competition and no competition were replicated 12 times, as follows: (1) one seedling of *I. cairica* per pot, indicated by Ic; (2) one seedling of *P. lobata* per pot, indicated by Pl; (3) one seedling of *P. scandens* per pot, indicated by Ps; (4) two seedlings of I. cairica per pot, indicated by Ic(Ic); (5) two seedlings of *P. lobata* per pot, indicated by Pl(Pl); (6) two seedlings of *P. scandens* per pot, indicated by Ps(Ps); (7) one seedling of *I. cairica* and one of *P. lobata* per pot, indicated by Ic(Pl) or Pl(Ic), Ic(Pl) means *I. cairica* growing with *P. lobata* under interspecific competition and Pl(Ic) means *P. lobata* growing with *I. cairica* under interspecific competition; (8) one seedling of *I. cairica* and one of *P. scandens* per pot, indicated by Ic(Ps) or Ps(Ic), Ic(Ps) means *I. cairica* growing with *P. scandens* under interspecific competition and Ps(Ic) means *P. scandens* growing with *I. cairica* under interspecific competition; (9) one seedling of *P. lobata* and one of *P. scandens* per pot, indicated by Pl(Ps) or Ps(Pl), Pl(Ps) means *P. lobata* growing with *P. scandens* under interspecific competition and Ps(Pl) means *P. scandens* growing with *P. lobata* under interspecific competition.

Pots were watered when plants showed signs of drought stress, and were randomly moved every week to ensure that all the plants were growing under the same environmental conditions. A pergola was constructed for the plants to climb as they grew up. The average monthly temperatures during the experimental period, March to July 2009, were 20.2–28.8 °C.

### Measurements of chlorophyll fluorescence parameters in a pot experiment

In June 2009, two months after transplanting, chlorophyll fluorescence parameters were determined on a clear sunny day. Specifically, they were first measured *in situ* with a portable fluorimeter PAM-2100 (Walz, Germany) on June 21, 2009. All fluorescence measurements were started after an additional 20-min dark adaptation. The maximal photochemical efficiency of PSII (*F*_*v*_/*F*_*m*_) was calculated as *F*_*v*_/*F*_*m*_ = (*F*_*m*_ − *F*_o_)/*F*_m_^34^. *F*_*m*_ means maximal fluorescence yield of a dark-adapted leaf and *F*_*o*_ means minimum fluorescence yield of a dark-adapted leaf. The steady-state (*F*_*s*_) and maximum fluorescence (*F*_m_′) in the light-adapted state were measured under actinic light at a photosynthetic photon flux density (PPFD) of 800 μmol m^−2^ s^−1^. The effective photochemical efficiency of PSII (*Φ*_*PSII*_) was calculated as *Φ*_*PSII*_ = (*F*_*m*_′− *F*_*s*_)/*F*_*m*_′^35^. Total electron transport rate (ETR) through PSII was estimated according to^36^: ETR = *Φ*_*PSII*_ × PPFD × *a* × 0.5, where *a* is the leaf absorption that is estimated as 0.84. The factor 0.5 was based on the assumption of an equal distribution of photons between PSI and PSII. Incident PPFD was measured with a quantum sensor^35^. Rubisco content of specific leaf area was estimated as Rubisco (g m^−2^) = ETR × 0.014^37^.

### Measurements of gas exchange parameters in a pot experiment

At the same time as chlorophyll fluorescence measurements were made, gas exchange parameters were determined using the LI 6400 portable gas exchange system (LI-COR Inc., Lincoln, NB, USA). Measurements commenced at 8:00 a.m. and were completed within 2 h in full sunshine. PPFD of the natural light ranged from 800 to 1000 μmol m^−2^ s^−1^, ambient temperature ranged from 28 to 30 °C. CO_2_ concentration inside the leaf chamber was maintained at 380 cm^3^ m^−3^ through the CO_2_-controlling system of the LI-6400 attached to a portable CO_2_ cylinder. The PPFD of 800 μmol m^−2^ s^−1^ on the cuvette surface was provided by an LED source. Before taking readings, leaves were equilibrated under the artificial light conditions in the leaf chamber for at least 10 min. During measurements, the relative air humidity was about 75% and leaf temperature was maintained at 25 °C. Net photosynthetic rate (*P*_*n*_), intercellular CO_2_ concentration (*C*_*i*_), stomatal conductance (*G*_*s*_) and transpiration rate (*T*_*r*_) were recorded. The stomatal limitation (*L*_*s*_) was estimated as *L*_*s*_ = 1 − *C*_*i*_/*C*_*a*_, where *C*_*a*_ is the atmospheric CO_2_ concentration^38^,^39^,^40^.

### Plant growth measurements in a pot experiment

In July 2009, when flowers started to appear, plants were harvested. After removing the cutting fragments of *I. cairica* rhizomes, the leaves, stems, and roots were separated from each plant and dried to a constant weight for at least 48 h at 60 °C and then weighed. The total biomass was the sum of leaves, stems and roots. Root mass ratio (RMR) was calculated as the biomass of root in proportion to the total biomass. Caulis length was measured with a roll ruler.

To test if the two native species had a competitive ability superior to the alien, we considered both the native competition and the alien impact. First, with regard to the native competition, we tested whether the effects of the two natives on the alien were larger than (a) the effect of the alien on the natives and (b) the effects between the natives. Second, focusing on the alien impact we tested if the effect of the alien on the two natives was lower than the effects between the natives. A relative interaction index (RII) has been proposed by Armas *et al.*^21^ to estimate the intensity of the effect of competition. RII is expressed as: RII = (*B*_*w*_ − *B*_*o*)_/(*B*_*w*_ + *B*_*o*_), where *B*_*w*_ is the observed mass of the target plant when growing with another plant and *B*_*o*_ is the mean mass achieved by the target plant in the absence of intra- or inter-specific interaction^21^. This index has revealed several advantages compared to other competition intensity indices such as the relative competition intensity^41^,^42^. The RII of a target plant ranges from −1 for a plant completely out-competed by another plant to +1 for a plant facilitated by another plant so much that its biomass under only intraspecific interaction is negligibly small by comparison. When interspecific interaction and intraspecific interactions have equal effects on the biomass of a target plant, according to our definition in Methods, RII = 0. A negative value indicates competition (i.e., growth of the target species is reduced) and a positive value indicates facilitation (i.e., growth of the target species is promoted). Considering the fact that plants always grow as a population and not as an individual, here we modify the definition of RII slightly, such that *B*_*o*_ is the mean mass achieved by the target plant under intraspecific competition. In this definition of *B*_*o*_, RII = 0 when the interspecific interaction is identical with intraspecific interaction; it equals −1 for a plant completely out-competed by another plant, and equals +1 for a plant facilitated by another plant so much that its biomass in the presence of only intraspecific interaction is negligible by comparison.

### Establishment of a natural population of *I. cairica* in field

In April 2010, an experimental field was constructed because the real field with *I. cairica* growing naturally is always bumpy and it is difficult to measure the distribution density of the plant. Nine plots (1 m × 2 m) were established and located in the experimental station of The Vegetable Research Institute, Guangdong Academy of Agricultural Sciences, China (28^o^08′N, 113^o^09′E, 65 m a.s.l.). The gardening soil contained 12.6 g kg^−1^ of soil organic matter (SOM), 1.37 g kg^−1^ of total nitrogen (TN), 44.3 mg kg^-1^ of NO_3_-N, 8.12 mg kg^-1^ of NH_4_-N, 1.05 g kg^−1^ of total phosphorus (TP) and 25.9 mg kg^-1^ of available phosphorus (AP). About 21 regenerated plantlets of *I. cairica* cuttings were planted in each plot (1 m × 2 m) and the growing row spacing was 25–30 cm. The plantlets were watered in the first two weeks. Afterwards no water was added and the plants grew naturally. A 4-m high pergola was built for the plants to climb as they grew up. This gave simulated a natural population of *I. cairica*.

### Replacement-control treatments in the field

In April 2011, one year after *I. cairica* had been growing, the plants aboveground and most roots in 9 plots were cleared to mimic the real situation when the plants were weeded out artificially. Of the 9 plots, 3 plots were used to sow seeds of *P. lobata* (63 seeds/2 m^2^), 3 plots to sow seeds of *P. scandens* (63 seeds/2 m^2^), and the remaining 3 plots as controls (no seeds were sown). Prior to sowing, the seeds of *P. lobata* and *P. scandens* were soaked in water for 3 hours in order to increase the sprouting rate. The field was watered once a day after sowing until the seedlings rose up. The seedlings were thinned to 21 plants/2 m^2^ plot when they grew up to 20 cm high. After that, no water was added and the plants grew naturally. Approximately six months after replacement-control treatments, gas exchange parameters, plant biomass and soil chemical characteristics were measured.

### Gas exchange, plant growth and soil characteristics measurements in the field

Gas exchange measurements were measured on August 14, 2011. Procedures followed those of the pot experiment.

In September 2011, the plants were harvested. The leaves, stems, roots and flowers were separated from each plant and dried to a constant weight for at least 48 h at 60 °C and then weighed. The total biomass was the sum of leaves, stems, roots and flowers. Caulis length was measured with a roll ruler.

In addition, the surface soil (0-10 cm) in each plot was collected and soil chemical characteristics were measured. The soil organic matter (SOM) was determined using a K_2_Cr_2_O_7_-H_2_SO_4_ oxidation method, total nitrogen (TN) was measured using the Kjeldahl method, and the available NH_4_-N and NO_3_-N were determined in fresh soil samples through steam distillation^43^.

### Statistical analysis

All statistical tests were performed using SPSS 11.5 software (SPSS Inc., USA). Plant biomass variables, gas exchange parameters, the fluorescence variables and soil chemical characteristics were compared using one-way ANOVA, followed by least significant difference (LSD) tests at *P* < 0.05. All observations are independent of one another and scores in groups are normally distributed. A univariate *F*-test for each variable was used to interpret the respective effects. The equality of error variances was tested by using Levene’s test and the error variance of the dependent variable was considered to be equal across groups when *P* > 0.05.

## Additional Information

**How to cite this article**: Li, Weihua. *et al.* A new strategy for controlling invasive weeds: selecting valuable native plants to defeat them. *Sci. Rep.* **5**, 11004; doi: 10.1038/srep11004 (2015).

## Figures and Tables

**Table 1:** Changes in biomass of *I. cairica*, *P. lobata* and *P. scandensis* in four treatments: growing alone, under intraspecific competition, and under interspecific competition (mean ± SD, n = 12).

	**Total biomass (g)**	**Caulis length (m)**	**Root mass ratio**
Ic*	6.17 ± 0.82 f**	2.70 ± 0.39 a	0.26 ± 0.05 ab
Ic(Ic)	3.43 ± 0.66 h	2.54 ± 0.38 ab	0.17 ± 0.05 d
Ic(Ps)	3.34 ± 0.95 h	1.78 ± 0.42 f	0.25 ± 0.07 ab
Ic(Pl)	1.93 ± 0.50 i	1.80 ± 0.33 f	0.21 ± 0.06 bc
Pl	17.92 ± 1.51 a	2.41 ± 0.40 bc	0.20 ± 0.04 bc
Pl(Pl)	8.88 ± 0.73 d	1.26 ± 0.24 g	0.21 ± 0.04 bc
Pl(Ps)	11.11 ± 2.48 c	2.14 ± 0.32 cde	0.17 ± 0.03 d
Pl(Ic)	13.41 ± 2.03 b	2.26 ± 0.45 bcde	0.15 ± 0.04 de
Ps	7.33 ± 1.19 e	2.38 ± 0.31 bcd	0.32 ± 0.14 a
Ps(Ps)	5.56 ± 0.70 gf	2.11 ± 0.27 de	0.29 ± 0.03 ab
Ps(Pl)	4.53 ± 1.43 g	2.13 ± 0.32 cde	0.31 ± 0.13 a
Ps(Ic)	5.97 ± 1.35 f	2.01 ± 0.33 ef	0.31 ± 0.08 a

^*^Note: Ic means *I. cairica* growing alone; Ic(Ic) means *I. cairica* under intraspecific competition; Ic(Ps) means *I. cairica* growing with *P. scandens* under interspecific competition; Ic(Pl) means *I. cairica* growing with *P. lobata* under interspecific competition; Pl means *P. lobata* growing alone; Pl(Pl) means *P. lobata* under intraspecific competition; Pl(Ps) means *P. lobata* growing with *P. scandens* under interspecific competition; Pl(Ic) means *P. lobata* growing with *I. cairica* under interspecific competition; Ps means *P. scandens* growing alone; Ps(Ps) means *P. scandens* under intraspecific competition; Ps(Pl) means *P. scandens* growing with *P. lobata* under interspecific competition; Ps(Ic) means *P. scandens* growing with *I. cairica* under interspecific competition.

^**^Different letters within the same column indicate significant differences between competition treatments according to the Least-Significant Difference test (LSD-test, *P* < 0.05).

**Table 2:** Changes in chlorophyll fluorescence parameters (means ± SD, n_1_ = 5, n_2_ = 6).

**Plants**	***Fv/Fm***	**ETR**	***Φ*_*PSII*_**	**Rubisco content (g.m^−2^)**	**Stomatal limitation**
Ic*	0.79 ± 0.02 a**	29.5 ± 1.55 a	0.66 ± 0.02 a	0.32 ± 0.02 a	0.29 ± 0.06 ab
Pl	0.79 ± 0.01 a	28.8 ± 1.17 a	0.63 ± 0.03 ab	0.32 ± 0.01 a	0.37 ± 0.03 a
Ps	0.76 ± 0.02 b	28.3 ± 1.02 a	0.63 ± 0.02 ab	0.31 ± 0.01 a	0.24 ± 0.06 b
Ic(Ps)	0.76 ± 0.02 b	24.3 ± 1.39 b	0.56 ± 0.05 bc	0.27 ± 0.02 b	0.26 ± 0.06 b
Ic(Pl)	0.76 ± 0.02 b	25.3 ± 3.08 b	0.58 ± 0.08 c	0.28 ± 0.03 b	0.27 ± 0.12 b
Ic(Ic)	0.80 ± 0.01 a	29.2 ± 0.89 a	0.65 ± 0.02 a	0.32 ± 0.01 a	0.28 ± 0.06 b

^*^Ic, Pl and Ps means *I. cairica*, *P. lobata* and *P. scandens*, respectively, growing alone; Ic(Ps) and Ic(Pl) means *I. cairica* growing with *P. scandens* and *P. lobata*, respectively, under interspecific competition; Ic(Ic) means *I. cairica* under intraspecific competition. *F*_*v*_/*F*_*m*_ means the maximal photochemical efficiency of PSII, ETR means total electron transport rate, and *Φ*_*PSII*_ means the effective photochemical efficiency of PSII.

^**^Different letters within the same column indicate significant differences between competition treatments according to Least-Significant Difference test (LSD-test, *P* < 0.05).

**Table 3:** Changes in plant biomass and caulis length in the plots (means ± SD, n = 3, Size of each plot = 2. 0 m^2^).

**Biomass**	**Total (kg)**	**Roots (kg)**	**Stems (kg)**	**Leaves (kg)**	**Flowers (kg)**	**Caulis length (m)**
Ic(CK)*	9.70 ± 1.35 b**	1.51 ± 0.29 a	4.03 ± 0.30 b	4.11 ± 0.77 b	0.056 ± 0.010 a	2.54 ± 0.09 a
Ic(Pl)	2.25 ± 0.26 d	0.40 ± 0.09 b	1.07 ± 0.09 c	0.78 ± 0.19 c	0.007 ± 0.001 b	2.17 ± 0.10 c
Ic(Ps)	2.18 ± 0.16 d	0.38 ± 0.07 b	0.64 ± 0.05 c	1.14 ± 0.07 c	0.012 ± 0.003 b	2.33 ± 0.08 b
Pl(Ic)	15.31 ± 1.96 a	0.39 ± 0.06 b	5.75 ± 0.78 a	9.17 ± 1.22 a		2.44 ± 0.16 ab
Ps(Ic)	6.84 ± 1.30 c	0.51 ± 0.08 b	3.50 ± 0.85 b	2.83 ± 0.84 b		2.50 ± 0.09 a

^*^Ic(CK) means *I. cairica* recruiting in the control plots not replaced by any native species; Ic(Pl) means *I. cairica* recruiting in the plots replaced by the native species *P. lobata*; Ic(Ps) means *I. cairica* recruiting in the plots replaced by the native species *P. scandens*; Pl(Ic) means *P. lobata* in the same plots as Ic(Pl); Ps(Ic) means *P. scandens* in the same plots as Ic(Ps).

^**^Different letters within the same column indicate significant differences between replacement control treatments according to the Least-Significant Difference test (LSD-test, *P* < 0.05).

**Table 4:** Changes of soil chemical characteristics in the field plots (means ± SD, n = 3).

	**Total nitrogen (g kg^−1^)**	**NH_4_-N (mg kg^−1^)**	**NO_3_-N (mg kg^−1^)**	**Soil organic matter (g kg^−1^)**
Ic(CK)*	0.43 ± 0.14 c	3.33 ± 0.70 c	33.05 ± 8.09 b	8.64 ± 1.03 b
Pl	4.22 ± 1.07 a	5.19 ± 0.47 a	48.30 ± 5.58 a	12.66 ± 0.80 a
Ps	1.55 ± 0.36 b	4.08 ± 0.33 b	36.65 ± 9.36 b	12.61 ± 1.05 a

^*^Ic(CK) means *I. cairica* recruiting in the control plots not replaced by any native species; Pl means the plots replaced by the native species *P. lobata*; Ps means the plots replaced by the native species *P. scandens*;

^**^Different letters within the same column indicate significant differences between replacement control treatments according to the Least Significant Difference test (LSD-test, *P* < 0.05).

## References

[b1] Bhattacharjee J., Taylor J. P., Smith L. M. & Haukos D. A. Seedling competition between native cottonwood and exotic saltcedar: implications for restoration. Biol. Invasions 11, 1777–1787 (2009).

[b2] Knochel D. G., Flagg C. & Seastedt T. R. Effects of plant competition, seed predation, and nutrient limitation on seedling survivorship of spotted knapweed (*Centaurea stoebe*). Biol. Invasions 12, 3771–3784 (2010).

[b3] Sheley R. L., Jacobs J. S. & Carpinelli M. L. [Spotted knapweed]Biology and management of noxious rangeland weeds [ Sheley R. L. & Petroff J. K. (eds)] [350–361] (Oregon State University, Cornallis, 1999).

[b4] Brockerhoff E. G., Withers T. M., Kay M. & Faulds W. [Impact of the defoliator *Cleopus japonicus* (Coleoptera: Curculionidae) on *Buddleja davidii*in the laboratory] Proc.52nd NZ Plant Prot. Conf. [113–118] (New Zealand Plant Protection Society, Auckland, 1999).

[b5] CAB International Institute of Biological Control. Digest: potential for biological control of Cuscuta spp. and Orobanche spp. Biocontrol News Info. 8, 193–199 (1987).

[b6] Watt M. S., Whitehead D., Kriticos D. J., Gous S. F., & Richardson B. Using a process-based model to analyse compensatory growth in response to defoliation: Simulating herbivory by a biological control agent. Biol. Control 43, 119–129 (2007).

[b7] Li M. G. *et al.* Evaluation of the controlling methods and strategies for *Mikania micrantha* H. B. K. Acta Ecol. Sin. 32, 3240–3251 (2012).

[b8] Yin Z. Y. *et al.* A preliminary study on ecological control of *Mikania micrantha* H. B. K. Guangdong Forest. Sci. Tech. 19, 17–22 (2003).

[b9] Lu C. Y., Hu H. Y., Zhang M. Q., Zhong Y. T. & Zheng F. Z. Studies on the control of alien invasive plant Macfadyena unguis-cati with ecological substitution. Plant Prot. 31, 53–56 (2005).

[b10] Funk J. L., Cleland E. E., Suding K. N. & Zavaleta E. S. Restoration through reassembly: plant traits and invasion resistance. Trends Ecol. Evol. 23, 695–703 (2008). 18951652 10.1016/j.tree.2008.07.013

[b11] Price J. N. & Partel M. Can limiting similarity increase invasion resistance? A meta-analysis of experimental studies. Oikos 122, 649–656 (2013).

[b12] Kimball S., Lulow M. E., Mooney K. A. & Sorenson Q. M. Establishment and management of native functional groups in restoration. Restor. Ecol. 22, 81–88 (2014).

[b13] Fang R. Z. & Staples G. W. [Convolvulaceae]. Flora of China Vol. 16 [ Wu C. Y. & Raven P. (eds)] [271–325] (Science Press and Missouri Botanical Garden Press, Beijing and St. Louis, MO, 1995).

[b14] Li Z. Y. & Xie Y. Invasive Alien Species in China [136] (China Forestry Publishing House, Beijing, 2002).

[b15] Wu Y. Q. & Hu Y. J. Researches on photosynthetic characteristics of exotic plants *Wedelia trilobata*, *Pharbitis nil* and *Ipomoea cairica*. Acta Ecol. Sin. 24, 2334–2339 (2004).

[b16] Shao Z. F., Zhao H. B., Qiu S. S., Yang Y. B. & Peng S. L. Study on the most harmful exotic plants in Shenzhen city. Ecol. Environ. 15, 587–593 (2006).

[b17] Qiu D. P., Huang D. C. & Zhuang W. S. Analysis of four most harmful external invasion plants in Jieyang city. *Acta Agric*. Jiangxi 19, 36–37 (2007).

[b18] European and Mediterranean Plant Protection Organization (EMPPO). Bull. OEPP/EPPO Bull. 37, 230–235 (2007).

[b19] Xiong Z. & Lang J. Pharmacological actions and clinical applications of *Paederia scandens* (Lour.) Merr. China Mod. Doctor 50, 27–29 (2012).

[b20] Aerts R. Interspecific competition in natural plant communities: mechanisms, trade-offs and plant-soil feedbacks. J. Exp. Bot. 50, 29–37(1999).

[b21] Armas C., Ordiales R. & Pugnaire I. Measuring plant interactions: a new comparative index. Ecology 85, 2682–2686 (2004).

[b22] Gaudent C. L. & Keddy P. A. Comparative approach to predicting competitive ability from plant traits. Nature 34, 242–243 (1988).

[b23] Keddy P., Nielsen K., Weiher E. & Lawson R. Relative competitive performance of 63 species of terrestrial herbaceous plants. J. Veg. Sci. 13, 5–16 (2002).

[b24] Domènech R. & Vilà M. Response of the invader *Cortaderia selloana* and two coexisting natives to competition and water stress. Biol. Invasions 10, 903–912 (2008).

[b25] Anderson J. M., Chow W. S. & Goodchild D. J. Thylakoid membrane organisation in sun/shade acclimation. Aust. J. Plant Physiol. 15, 11–26 (1988).

[b26] Peng H., Gui F. R., Li Z. Y., Li J. & Wan F. H. Competition effect of *Imperata cylindrical* to *Ageratina adenophora*. Chinese J. Ecol. 29, 1931–1936 (2012).

[b27] Fargione J., Brown C. S. & Tilman D. Community assembly and invasion: an experimental test of neutral versus niche processes. Proc. Natl. Acad. Sci. USA. 100, 8916–8920 (2003). 12843401 10.1073/pnas.1033107100 PMC166413

[b28] Emery S. M. Limiting similarity between invaders and dominant species in herbaceous plant communities? J. Ecol. 95, 1027–1035 (2007).

[b29] Adler P. B., Hillerislambers J. & Levine J. M. Weak effect of climate variability on coexistence in a sagebrush steppe community. Ecology 90, 3303–3312 (2009). 10.1890/08-2241.1 20120800

[b30] Larson D. L. *et al.* Using prairie restoration to curtail invasion of Canada thistle: the importance of limiting similarity and seed mix richness. Biol. Invasions 15, 2049–2063 (2013).

[b31] Roberts R. E., Clark D. L. & Wilson M. V. Traits, neighbors, and species performance in prairie restoration. App. Veg. Sci. 13, 270–279 (2010).

[b32] Weltzin J. F., Muth N. Z., Von Holle B. & Cole P. G. Genetic diversity and invasibility: a test using a model system with a novel experimental design. Oikos 103, 505–518 (2003).

[b33] Byun C., Blois S. & Brisson J. Plant functional group identity and diversity determine biotic resistance to invasion by an exotic grass. J. Ecol. 101, 128–139 (2013).

[b34] Schreiber U., Schliwa U. & Bilger W. Continuous recording of photochemical and nonphoto chemical chlorophyll fluorescence quenching with a new type of modulation fluorometer. Photosynth. Res. 10, 51–62 (1986). 24435276 10.1007/BF00024185

[b35] Genty B., Briantais J. M. & Baker N. R. The relationship between the quantum yield of photosynthetic electron transport and quenching of chlorophyll fluorescence. Biochim. Biophys. Acta 990, 87–92 (1989).

[b36] Krall J. P. & Edward G. E. Relationship between photosystem II activity and CO_2_ fixation in leaves. Physiol. Plantarum 86, 180–187 (1992).

[b37] Evans J. R. & Porter H. Photosynthetic acclimation of plants to growth irradiance: the relative importance of species leaf area and nitrogen. Plant Cell Environ. 24, 755–767 (2001).

[b38] Berry J. A. & Downton W. S. [Environmental regulation of photosynthesis]. Photosynthesis Vol. II [ Govindjee N. Y. (ed.)] [263–343] (Academic Press, New York, 1982).

[b39] Zou Q. Studies on physiological drought resistance of crop [155–242] (Shandong Sci. Tech. Press, Jinan, 1994).

[b40] Shao X. W., Han M., Han Z. M., Kong W. W. & Yang L. M. Relationship between diurnal changes of photosynthesis of *Scutellaria baicalensis* and environmental factors in different habitats. Acta Ecol. Sin. 29, 1470–1477 (2009).

[b41] Grace J. B. On the measurement of plant competition intensity. Ecology 76, 305–308 (1995).

[b42] Goldberg D. E., Rajaniemi T., Gurevitch J. & Stewart-Oaten A. Empirical approaches to quantifying interaction intensity: competition and facilitation along productivity gradients. Ecology 80, 1118–1131 (1999).

[b43] Institute of Soil Science, the Chinese Academy of Science. Physical-Chemical Analysis of Soil. [146–148] (Shanghai Sci. Tech. Press, Shanghai, China, 1978).

